# Effects of Dignity Therapy for Palliative Care Patients and Family Caregivers: A Systematic Review

**DOI:** 10.7759/cureus.70431

**Published:** 2024-09-29

**Authors:** Sara H Haneef, Marwah Abdullah

**Affiliations:** 1 Family Medicine, King Abdulaziz University Hospital, Jeddah, SAU; 2 Family Medicine, King Abdul-Aziz Medical City, Ministry of National Guard, Riyadh, SAU

**Keywords:** anxiety, dignity therapy, palliative care, quality of life, stress

## Abstract

Dignity Therapy (DT) is a psychosocial intervention aimed at alleviating existential distress, enhancing meaning, and improving the overall well-being of palliative care patients and their family caregivers. While individual studies have demonstrated the benefits of DT, a comprehensive evaluation of its impact on key outcomes such as quality of life (QoL), depression, anxiety, and well-being is needed. This study aimed to systematically review the effects of DT on palliative care patients and their family caregivers, focusing on outcomes related to QoL, psychological distress (depression and anxiety), and overall well-being.

A systematic review was conducted by searching multiple databases, including Cochrane Library, PubMed, ScienceDirect, Web of Science, and Scopus, for randomized controlled trials (RCTs) evaluating DT in palliative care. Inclusion criteria were RCTs reporting on QoL, depression, anxiety, well-being, and dignity-related distress. Two reviewers independently screened studies and extracted data. Study quality was assessed using the Cochrane Risk of Bias tool.

Eight RCTs met the inclusion criteria. DT demonstrated significant improvements in QoL and well-being among palliative care patients, with reductions in depression and anxiety. Improvements were observed in the physical, psychological, and social domains of QoL. Family caregivers also experienced enhanced spiritual well-being, hope, family cohesion, and adaptability. However, not all outcomes were consistently improved across all studies, indicating variability in DT effectiveness.

DT is a promising intervention for enhancing QoL and reducing psychological distress among palliative care patients and their family caregivers. While evidence supports its beneficial impact, further high-quality research is required to confirm these findings and clarify the mechanisms through which DT works. Incorporating DT into palliative care practice is recommended to address the comprehensive needs of patients and their families.

## Introduction and background

Dignity therapy (DT), an innovative approach within palliative care, has gained prominence for its potential to address the complex psychological and existential needs of terminally ill patients and their family caregivers. Developed by Dr. Harvey Max Chochinov, DT aims to enhance the quality of life (QoL) by helping individuals reflect on their personal values, life experiences, and legacy, thereby fostering a sense of meaning and continuity [1]. This intervention is predicated on the understanding that maintaining dignity is a critical component of quality end-of-life care. In recent years, DT has been the subject of extensive research, highlighting its significance in improving emotional well-being and reducing psychological distress among patients in palliative care. Studies have shown that this therapy can significantly impact patients by providing them with an opportunity to engage in meaningful reflection, which can help alleviate feelings of hopelessness and despair [2]. This is particularly relevant as individuals facing terminal illnesses often experience a profound sense of loss and anxiety about their legacy and the impact of their lives.

The benefits of DT extend beyond the patients themselves; they also encompass their family caregivers [3]. Caregivers frequently experience emotional strain and distress due to the demanding nature of their role and the impending loss of their loved one. Research has indicated that DT can provide significant emotional support to caregivers by facilitating open communication and helping them cope with the emotional burdens associated with end-of-life care. The impact of DT on the therapeutic relationship between patients and caregivers is an important consideration. The therapy encourages dialogue and reflection, fostering a deeper understanding between patients and their caregivers. This improved communication can lead to a more compassionate and supportive care environment, which is crucial in palliative settings where emotional and psychological needs are paramount. As evidenced by research conducted, the positive effects of DT on communication and emotional support contribute to a more holistic approach to end-of-life care, addressing not just the physical but also the psychological and emotional aspects of the patient and caregiver experience [4]. Despite its benefits, DT utilization is not widespread. According to Chochinov et al., the impact of DT depends primarily on factors such as the severity of the illness and the patient's pre-existing psychological state [5]. Currently, research is lacking on the implementation of DT, including variations in its effectiveness based on patient demographics and the specific context of palliative care settings. Therefore, while DT is a valuable tool, it may require adaptation to meet the diverse needs of different patient populations.

Therefore, DT represents a significant advancement in palliative care, offering valuable support to terminally ill patients and their family caregivers. By facilitating meaningful reflection and communication, DT helps address critical psychological and emotional needs, enhancing the overall QoL and providing a sense of purpose and continuity [6]. However, ongoing research and adaptation are essential to fully realize its potential and address the challenges associated with its implementation. This systematic review aims to explore the impact of DT on both palliative care patients and their family caregivers, examining its effectiveness in enhancing QoL and psychological well-being, providing emotional and spiritual support, and addressing anxiety, depression and existential distress. By synthesizing evidence from various studies, this review aims to elucidate the benefits and limitations of DT, providing insights into its role in improving the holistic care of individuals during the end-of-life phase.

## Review

Materials and methods

Definition of Outcomes

This systematic review followed the Preferred Reporting Items for Systematic Reviews and Meta-Analyses (PRISMA) guidelines. The review aimed to evaluate the effects of DT on palliative care patients and their family caregivers, focusing on several key outcomes: QoL, depression, anxiety, well-being, and dignity-related distress. QoL was assessed using validated instruments such as the EQ-5D (5-level version) and the SF-36 (36-item version). The EQ-5D evaluates mobility, self-care, usual activities, pain/discomfort, and anxiety/depression, while the SF-36 measures eight domains, including physical functioning and emotional role functioning. Cutoff thresholds for inclusion were based on studies using these scales, where significant changes (e.g., an improvement of at least 10% in physical and mental component summaries) were considered clinically relevant. Depression was measured using the Beck Depression Inventory (BDI-II) and the Hospital Anxiety and Depression Scale (HADS). For the BDI-II, scores ≥14 indicated moderate depression, while scores ≥11 on the HADS were considered significant for anxiety or depression. Anxiety was similarly evaluated using the HADS, with a threshold of 8 or above signaling clinically relevant anxiety. Well-being was assessed using the Warwick-Edinburgh Mental Well-being Scale (WEMWBS), with studies including participants scoring ≤40, which reflects low mental well-being. Dignity-related distress was evaluated using dignity scales specific to palliative care, such as the Patient Dignity Inventory (PDI), which covers existential, psychological, and social dimensions. Only studies using the full versions of these scales were included to ensure a comprehensive measurement of distress.

The inclusion criteria for selecting studies were comprehensive. Eligible studies included randomized controlled trials (RCTs) and controlled clinical trials (CCTs). Participants had to be palliative care patients and their family caregivers, with the intervention being DT as defined by the original study authors. Studies needed to report on at least one of the defined outcomes to be included. Only peer-reviewed articles published in English were considered, encompassing studies from inception to the search date. Exclusion criteria included reviews and case reports, duplicate publications, studies where data were not available or could not be extracted for the study groups, studies with insufficient or unclear data regarding outcomes, animal studies, qualitative studies, laboratory studies, posters, theses, protocols, and pilot studies

Search Strategy

A comprehensive search was conducted across multiple databases, including the Cochrane Library, PubMed, Science Direct, Web of Science, and Scopus, with the search restricted to studies published up to July 2024. The search strategy was designed to capture relevant literature by including terms such as (“palliative care” OR “patients” OR “family caregivers” OR “caregivers”) AND (“dignity therapy” OR “dignity intervention”) AND (“patient well-being” OR “quality of life” OR “caregiver burden” OR “effectiveness” OR “role” OR “impact”). In addition to database searches, references from selected articles and relevant review papers were manually reviewed to identify any further studies. Conference abstracts and prospective trial registries were also searched to ensure comprehensive coverage of the available literature.

Inclusion and Exclusion Criteria

The inclusion criteria for this review encompassed randomized controlled trials (RCTs) and controlled clinical trials (CCTs) that reported on at least one of the predefined outcomes. Eligible studies had to involve palliative care patients and their family caregivers, with DT as the intervention. Only peer-reviewed articles published in English were considered, including studies from inception to the search date in July 2024. Exclusion criteria included reviews, case reports, and duplicate publications. Studies with insufficient or unclear data, where outcomes were not extractable, were excluded, as were qualitative studies, animal studies, laboratory studies, posters, theses, protocols, and pilot studies. These parameters were applied to ensure the inclusion of studies with robust and relevant data for analysis.

Screening and Data Extraction

The screening process involved two stages. Initially, the two authors independently screened the titles and abstracts of the retrieved studies to assess their relevance. Any discrepancies between the authors were resolved through detailed discussion and mutual agreement. In the second stage, the full texts of potentially eligible studies were reviewed by both authors to confirm their eligibility based on the predefined inclusion criteria.

Data extraction was conducted using a standardized form to ensure consistency and completeness. Both authors performed the extraction independently and resolved any discrepancies through discussion and consensus. This approach maintained a high level of rigor and accuracy throughout the review process.

The standardized data extraction form used for this systematic review was meticulously designed to capture relevant information across a range of domains. The form included the following specific data points:

Study Identification: Authors, year of publication, and title of the study; journal name and study design (e.g., RCT, CCT); registration details and study period.

Study Characteristics: Total number of participants, divided into cases and control groups; age distribution (mean ± SD) and gender distribution (percentage of males and females); specific details regarding the intervention and control groups.

Outcome Measures: QoL assessments, including tools such as EQ-5D and SF-36, with mean scores and standard deviations reported; depression, anxiety, well-being, and dignity-related distress measures using tools like BDI, HADS, WEMWBS, and Patient Dignity Inventory; statistical results including mean differences, p-values, and confidence intervals.

Risk of bias Assessment: Random sequence generation, allocation concealment, blinding of participants and personnel, blinding of outcome assessment, incomplete outcome data, selective reporting, and other biases.

Results Summary: Main findings related to each outcome measure, including improvements or deteriorations in QoL, depression, anxiety, well-being, and dignity-related distress; statistical significance of changes and effect sizes where applicable.

Quality Assessment: Overall quality of the study as per the Cochrane Risk of Bias tool, with ratings for each bias domain.

Quality Assessment

To evaluate the risk of bias in the included studies, the Cochrane Risk of Bias tool was employed specifically for RCTs. This tool assesses several key domains of study quality. It examines random sequence generation to determine if the method used was adequate to ensure proper randomization. Allocation concealment is evaluated to ensure that the process of assigning participants to intervention or control groups was adequately concealed from those responsible for this assignment. The tool also assesses blinding of participants and personnel to ascertain whether they were blinded to the intervention being administered, and blinding of outcome assessment to verify if outcomes were assessed by individuals unaware of the participants' group assignments. Additionally, the handling of incomplete outcome data is reviewed to check if appropriate methods were employed to address any missing data. The tool also scrutinizes selective reporting to ensure that all planned outcomes were reported and that no results were selectively omitted. Finally, it identifies any other sources of bias that might affect the validity of the study results.

The quality assessment process was conducted independently by both authors to ensure a thorough evaluation. Discrepancies in the assessments were resolved through detailed discussions between the two reviewers. In cases where consensus could not be reached through discussion alone, the resolution was achieved through mutual agreement based on the study data and context. Due to the limited number of authors, no third-party reviewer was involved in the process.

Based on the assessments, studies were categorized into three levels of risk: low, moderate, or high. To further ensure the reliability of the review findings, a sensitivity analysis was conducted, excluding studies classified as having a high risk of bias. This approach aimed to reinforce the robustness of the conclusions drawn from the remaining studies.

Results

Search Results

**Figure 1:**
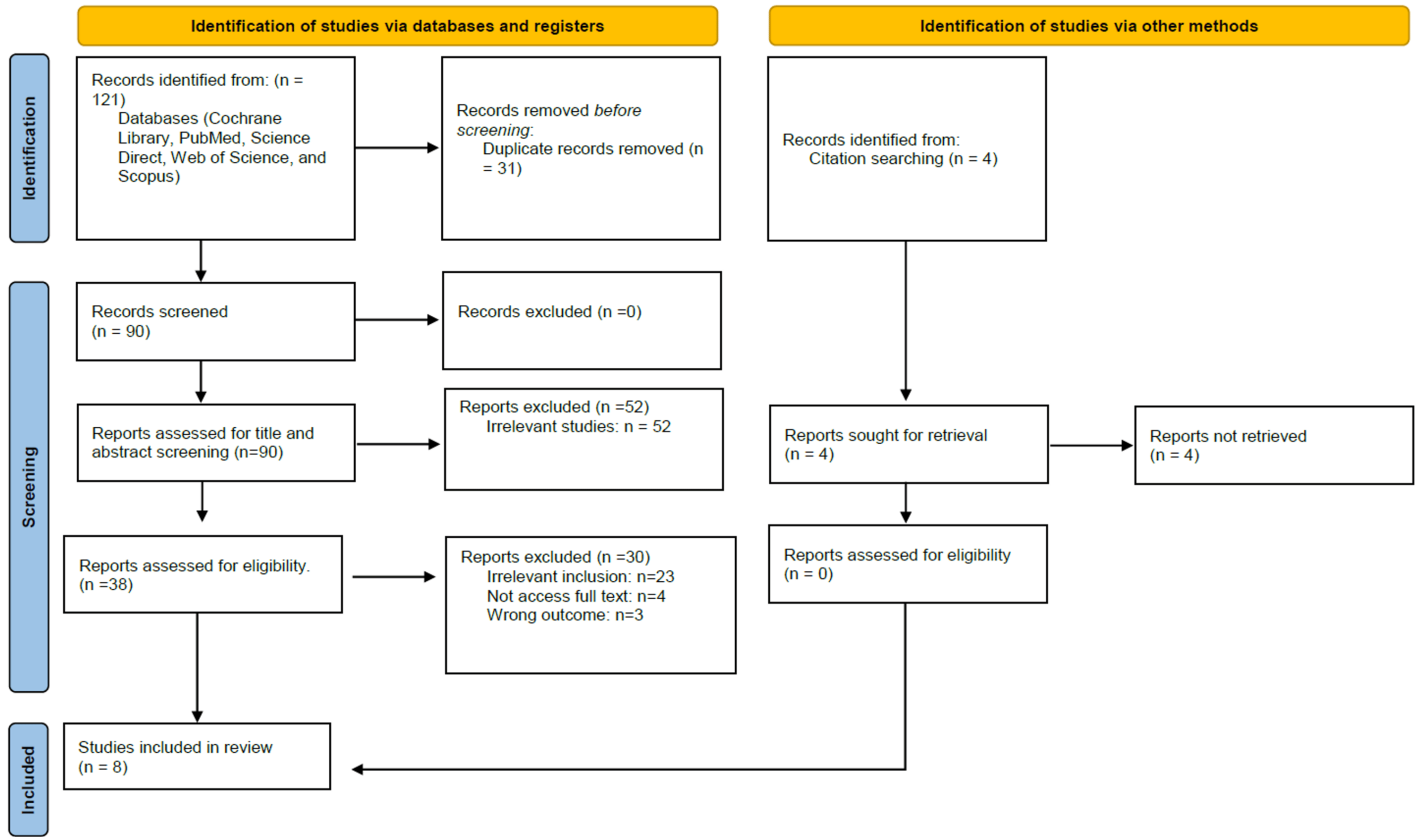
PRISMA flowchart PRISMA: Preferred Reporting Items for Systematic Reviews and Meta-Analyses

We were able to uncover a total of 121 citations using the previously specified search techniques, which were subsequently reduced to 90 after duplicates were eliminated. Only 38 citations remained after the title and abstract screening that qualified for the following stages. Only eight articles met our inclusion and exclusion criteria after the full-text screening [7-14]. Figure 1 displays the thorough search and screening procedure.

Results of Quality Assessment

Table 1 summarizes the risk of bias for the studies included in this review, as assessed using the Cochrane Risk of Bias tool for RCTs. The assessment covers several key domains: random sequence generation, allocation concealment, blinding of participants and personnel, blinding of outcome assessment, incomplete outcome data, selective reporting, and other biases.

**Table 1:** Cochrane Risk of Bias tool for RCTs

Study	Random sequence generation	Allocation concealment	Blinding of participants and personnel	Blinding of outcome assessment	Incomplete outcome data	Selective reporting	Other bias
Seiler et al. (2024) [7]	Low	Unclear	Unclear	Unclear	Low	Low	Low
Jenewein et al. (2021) [8]	Low	Low	High	Unclear	Low	Low	Low
Wang et al. (2021) [9]	Low	Low	Low	Low	Low	Low	Low
Iani et al. (2020) [10]	Low	Low	High	High	High	Low	Low
Karimi et al. (2020) [11]	Low	Unclear	High	Unclear	Low	Low	Low
Weru et al. (2020) [12]	Low	Low	High	High	Low	Low	Low
Juliao et al. (2014) [13]	Low	Low	Low	Low	Low	Low	Low
Chochinov et al. (2011) [14]	Low	Low	Unclear	Unclear	High	Low	Low

Overall, the majority of studies exhibit a low risk of bias in critical domains. Specifically, Seiler et al. [7] and Juliao et al. [13] show low risk in all assessed domains except for blinding of participants and personnel, where the risk is unclear in Seiler et al.'s study. This suggests that these studies are relatively robust with minimal bias affecting their outcomes. Jenewein et al. [8] also demonstrate a generally low risk of bias, with the exception of high risk in the blinding of participants and personnel. This limitation may influence the internal validity of the study results, potentially impacting the reliability of the reported outcomes. Wang et al. [9] and Karimi et al. [11] show low risk across most domains, although Karimi et al.'s study has unclear risk in allocation concealment and blinding of outcome assessment. The presence of unclear risk in these areas suggests that while the study has generally strong methodological rigor, there are areas where details were not sufficiently reported or controlled. Iani et al. [10] present a mixed picture with low risk in random sequence generation and allocation concealment but high risk in blinding of participants and personnel, as well as incomplete outcome data. These high-risk areas indicate significant methodological concerns that could affect the validity of the findings. Weru et al. [12] study is similar to Iani et al. [10] in that it has low risk in several domains but high risk in blinding of participants and personnel and blinding of outcome assessment. The high risk in these domains may compromise the objectivity of the outcomes and the overall strength of the evidence. Chochinov et al. [14] show a low risk of bias in most domains but has high risk related to incomplete outcome data. This high-risk area indicates potential issues with the completeness of data reporting, which may affect the study's conclusions.

In summary, while many studies exhibit low risk across critical domains, indicating generally strong methodological quality, there are notable exceptions where a high or unclear risk of bias could affect the reliability of the results. These variations in risk highlight the importance of critically evaluating methodological rigor when interpreting the findings of this review.

Characteristics of the Included Studies

This systematic review incorporated eight studies evaluating the effects of DT on palliative care patients and their family caregivers. The studies spanned from 2005 to 2024, representing a diverse array of geographic locations and methodological approaches. Table 2 summarizes the baseline characteristics of these studies, providing an overview of participant demographics, study design, and publication details.

**Table 2:** Baseline characteristics of the included studies NR: not reported

Author	Registration	Country	Study type	Year of Publication	Study period	Total participants	Cases	Control	Total participants Age (Years)	Gender (M/F)
Seiler et al. [7]	NCT02646527	Switzerland	RCT	2024	2015-2021	136	68	68	59.4 ±17.6	59%/41%
Jenewein et al. [8]	NCT03692988	Switzerland	RCT	2021	2019-2021	108	54	54	81.2 ±5.7	46%/62%
Wang et al. [9]	ChiCTR1900021433	China	RCT	2021	2019	106	53	53	49.4±12.3	57%/49%
Iani et al. [10]	NCT04256239	Italy	RCT	2020	2018-2020	35	15	20	75.1±10.7	14%/21%
Karimi et al. [11]	NR	Iran	RCT	2020	2019	76	38	38	47.1± 9.8	55%/15%
Weru et al. [12]	PACTR201604001447244	Kenya	RCT	2020	2016-2017	144	72	72	NR	NR
Juliao et al. [13]	ISRCTN34354086	Portugal	RCT	2014	2010-2013	80	41	39	66.1± 12.9	37%/43%
Chochinov et al. [14]	NCT00133965	Canada	RCT	2011	2005-2008	326	108	218	64.2 ±14.6	49.4%/50.6%

The studies included in this review were conducted in various countries, including Switzerland, China, Italy, Iran, Kenya, Portugal, and Canada, and employed RCT designs. The sample sizes ranged from 35 to 326 participants, with a total of 1,040 participants across all studies. The age of participants varied considerably, from a mean age of 47.1 years in Karimi et al. [11] to 81.2 years in Jenewein et al. [8]. The gender distribution also varied, with some studies reporting balanced male-to-female ratios, while others had a higher percentage of female participants. In terms of study periods, these studies covered a range of time frames, from as early as 2005 to as recently as 2021. For instance, Chochinov et al. [14] reported on a study period from 2005 to 2008, while Seiler et al. [7] covered data from 2015 to 2021. The studies employed various methods of data collection, including both prospective and retrospective approaches. Tables 3-5 provide a detailed summary of the outcomes related to DT as reported in the included studies.

**Table 3:** Effects of dignity therapy on quality of life and psychological outcomes DT: Dignity therapy, HADS: Hospital Anxiety and Depression Scale, PRISM: Pictorial Representation of Illness and Self Measure, QOL: quality of life, CI: confidence interval, PDI: Patient Dignity Inventory, FACIT-Pal: Functional Assessment of Chronic Illness Therapy Palliative Care, FACIT-SP: Functional Assessment of Chronic Illness Therapy Spiritual Well-Being, χ2: chi square, df: degrees of freedom; NR: Not reported

Author	Quality of Life (QOL)	Psychological Distress	Patient Satisfaction & Recommendations
Seiler et al. [7]	FACIT-Pal-14: 6.15 (2.33; 9.96), p=0.01	HADS: F = 4.33, df = 1, 82.9; p = 0.04	DT usefulness: 2.55±1.19
			Recommend to others: 2.54±1.45
Jenewein et al. [8]	QOL (Physical): 5.14(1.48 to 8.81), p=0.007	HADS (Immediate): −2.69(−4.40 to −0.99), p=0.003	NR
Wang et al. [9]	Positive effect on family cohesion and adaptability	Decreased symptoms: anxiety and depression	NR
Iani et al. [10]	Meaning: 12.3±3.8, Peace: 8.9±2.8	Psychological distress: 17.3±5.9	NR
Karimi et al. [11]	QOL: 69.61±12.71, p<0.001	NR	NR
Weru et al. [12]	NR	Anxiety (p = 0.059)	NR
Juliao et al. [13]	NR	Depression T2(day4): -4.00 [ - 6.00, - 2.00], p<0.0001	NR
		Anxiety T2(day4): -3.00 [ - 5.00, - 1.00], p<0.0001	
Chochinov et al. [14]	QOL: χ2 =14.520, p<0.001	Depression: χ2 =9.379, p=0.009	Feeling satisfied: χ2=29.583, p<0.001

**Table 4:** Effects of dignity therapy on depression, anxiety, and other psychosocial factors DT: Dignity therapy, PRISM: Pictorial Representation of Illness and Self Measure, PDI: Patient Dignity Inventory, FACIT-Pal: Functional Assessment of Chronic Illness Therapy Palliative Care, FACIT-SP: Functional Assessment of Chronic Illness Therapy Spiritual Well-Being, χ2: chi square; NR: Not reported

Author	Hope	Spiritual Well-being	Dignity	Existential and Coping
Seiler et al. [7]	NR	PRISM: DT + DT combined: 1.55 ±0.76, p	No significant change in dignity-related distress	NR
Jenewein et al. [8]	NR	FACIT-SP: 2.74 (0.06 to 5.42), p=0.045	PDI: −6.56(−9.83 to −3.29), p<0.001 (Immediate)	NR
Wang et al. [9]	NR	Enhanced spiritual well-being		NR
Iani et al. [10]	NR	Faith: 4.7±3.2	NR	Meaning and purpose: 4.5±4.2, Distress: 5.7±3.6
Karimi et al. [11]	Hope: 26.88±2.90, p=0.03	NR	NR	NR
Chochinov et al. [14]	NR	NR	Sense of dignity: χ2 =12.655, p=0.002	Existential distress: 22.7±6.5

**Table 5:** Detailed impact on specific psychological and quality of life measures DT: Dignity therapy, HADS: Hospital Anxiety and Depression Scale, PRISM: Pictorial Representation of Illness and Self Measure, QOL: quality of life, CI: confidence interval, PDI: Patient Dignity Inventory, FACIT-Pal: Functional Assessment of Chronic Illness Therapy Palliative Care, FACIT-SP: Functional Assessment of Chronic Illness Therapy Spiritual Well-Being

Author	Outcome Measures	Findings
Seiler et al. [7]	HADS, PRISM, DT usefulness, recommendation likelihood	Improvements in several measures including HADS and PRISM; majority of patients found DT useful.
Jenewein et al. [8]	HADS, PDI, QOL (physical, psychological, social, environment, overall), FACIT-SP	Significant improvements in immediate group across several QOL measures; less pronounced in delayed group.
Karimi et al. [11]	Hope, QOL	Significant improvement in hope and quality of life among the intervention group compared to control.
Chochinov et al. [14]	Depression, QOL, Well-being, Sense of Dignity	Significant reduction in depression and improvements in overall quality of life and well-being.

The findings reveal a range of effects on various psychological and quality-of-life outcomes. For instance, Seiler et al. [7] observed improvements in several domains, including functional and psychological well-being, with mean differences reported for Functional Assessment of Chronic Illness Therapy Palliative Care (FACIT-Pal)-14 and HADS scores. However, no significant differences were noted in overall psychological distress or spiritual well-being. Jenewein et al. [8] reported significant improvements in anxiety, physical QoL, and some aspects of psychological and environmental QoL for the immediate group, though the delayed group did not show the same level of benefit. Wang et al. [9] found positive effects on hope, spiritual well-being, and family cohesion, alongside reduced anxiety and depression symptoms. Iani et al. [10] observed mixed results, with some improvement in peace and meaning but no significant changes in existential distress or physical distress when comparing intervention to control groups. Karimi et al. [11] highlighted increases in hope and QoL, while Juliao et al. [13] reported significant reductions in depression and anxiety symptoms at multiple time points. Chochinov et al. [14] demonstrated improvements in QoL, well-being, and dignity, with significant findings related to overall satisfaction and perceived helpfulness of the intervention.

Discussion

This systematic review of DT for palliative care patients and their family caregivers has provided valuable insights into the effectiveness of this therapeutic approach. Overall, the results indicate that while DT can positively impact various aspects of patients' and caregivers' experiences, the effectiveness is variable across studies and outcomes. The analysis of the included studies demonstrates both the potential benefits and limitations of DT, reflecting a broad spectrum of outcomes that highlight the complex nature of palliative care interventions. This discussion synthesizes the findings from the reviewed studies, placing them within the broader context of existing literature to offer a comprehensive understanding of DT’s impact, effectiveness, and implications for future practice.

Quality of Life and Psychological Well-Being

QoL is a multifaceted concept that encompasses various dimensions of a person's well-being, including physical, psychological, social, and environmental aspects. In the context of palliative care, QoL often reflects the patient’s overall satisfaction with their health status, their ability to engage in daily activities, and their sense of personal fulfillment and comfort. The systematic review highlights that DT can significantly impact certain dimensions of QoL. For instance, Seiler et al. [7] and Jenewein et al. [8] observed improvements in QoL metrics such as the FACIT-Pal-14 and Pictorial Representation of Illness and Self Measure (PRISM) scores among patients receiving DT. These findings are consistent with broader literature suggesting that addressing existential concerns through DT can lead to enhanced QoL [14]. DT’s focus on personal legacy and life review may contribute to a sense of purpose and satisfaction, positively influencing patients' overall QoL. Wang et al. [9] reported improvements in hope, spiritual well-being, and family cohesion, which are integral components of QoL. These positive effects echo Chochinov et al.’s findings that DT can foster a sense of dignity and overall well-being [1]. The incorporation of spiritual and emotional support into palliative care reflects a growing recognition of these factors' importance in QoL [15,16]. DT’s potential to address existential and spiritual needs aligns with the literature emphasizing the value of holistic approaches in enhancing QoL for palliative care patients. Despite these positive outcomes, the review also highlights variability in DT’s impact on QoL. Iani et al. [10] and Karimi et al. [11] found mixed results, with some improvements in specific areas but not universally across all QoL dimensions. Iani et al.’s [10] study found enhancements in meaning and peace but noted no significant changes in existential distress or coping ability, suggesting that while DT can positively influence certain QoL aspects, its effectiveness is not uniformly experienced across all dimensions. This variability aligns with other research indicating that DT’s impact on QoL can vary depending on the specific aspects being targeted and individual patient needs [17]. The complexity of QoL means that while DT may improve certain aspects, it may not uniformly enhance all areas of patients' lives, reflecting the nuanced nature of QoL in palliative care settings.

Psychological well-being in palliative care encompasses various elements, including emotional states, coping abilities, and overall mental health [4]. It reflects how patients manage psychological distress, find meaning in their experiences, and maintain a sense of dignity and self-worth despite facing serious illness [18]. The review shows that DT can positively influence psychological well-being. For example, Juliao et al. [13] reported significant reductions in depression and anxiety, supporting DT’s effectiveness in improving emotional states. These findings are consistent with the literature suggesting that DT can be an effective strategy for alleviating psychological distress and enhancing emotional well-being [17]. Chochinov et al. [14] also observed reductions in depression and improvements in well-being, sense of dignity, and satisfaction with therapy. This highlights DT’s role in addressing emotional and existential concerns, contributing to an improved sense of psychological well-being. However, the review also reveals limitations in DT’s impact on psychological well-being. Weru et al. [12] reported minimal impact on anxiety, with results approaching statistical significance but not reaching it. This suggests that while DT may offer benefits in some areas, its impact on specific psychological symptoms like anxiety can be less pronounced. This observation is supported by literature including Li et al.’s qualitative exploration indicating that DT’s effects on certain symptoms can be variable and dependent on individual characteristics and intervention context [19]. The mixed results from Iani et al. [10] and Karimi et al. [11] further illustrate this variability. Iani et al. [10] found improvements in meaning and peace but no significant changes in coping ability, indicating that DT might not uniformly improve all dimensions of psychological distress [20]. Similarly, Karimi et al. [11] demonstrated significant improvements in hope and QoL, yet not all aspects of psychological distress were equally addressed by DT.

Emotional and Spiritual Support

Emotional and spiritual support are critical components of palliative care, aimed at addressing the profound psychological and existential needs of patients facing serious illness. This support helps patients navigate their emotional distress, find meaning in their experiences, and maintain a sense of dignity and peace during their end-of-life journey. DT is specifically designed to provide emotional and spiritual support by focusing on the patient's sense of dignity, life review, and legacy creation [6]. It addresses both psychological and existential needs, making it a valuable intervention in palliative care. The systematic review highlights DT’s effectiveness in providing emotional support. Studies such as those by Juliao et al. [13] demonstrate that DT can lead to significant reductions in depression and anxiety, indicating that DT effectively alleviates emotional distress. This aligns with broader research suggesting that DT can enhance emotional well-being by helping patients cope with psychological challenges and improve their overall mood [21,22]. DT’s role in providing spiritual support is evident in its impact on hope and spiritual well-being [23]. Wang et al. [9] found that DT improved patients' hope and spiritual well-being, which are integral aspects of spiritual support. This is consistent with the literature suggesting that DT helps patients find meaning and purpose in their lives, addressing their spiritual concerns [1]. By facilitating discussions about life’s meaning and legacy, DT helps patients address existential questions and enhances their spiritual well-being [23,24]. Despite the positive effects, the review also highlights some variability in DT’s effectiveness in providing emotional and spiritual support. For example, Seiler et al. [7] found improvements in certain measures but no significant changes in overall QoL or psychological distress, suggesting that DT may not uniformly address all emotional and spiritual needs. Similarly, Iani et al. [10] observed significant improvements in meaning and peace but no changes in existential distress or coping ability. This indicates that while DT can enhance specific aspects of emotional and spiritual well-being, its impact may not be uniform across all dimensions. Weru et al. [12] reported minimal impact on anxiety, indicating that DT may not be as effective in addressing certain emotional symptoms compared to others. This variability highlights the complexity of DT’s effects and suggests that its impact on specific emotional and spiritual aspects can differ based on individual patient characteristics and the context of the intervention [6,14]. The findings from this review are consistent with existing literature on the importance of emotional and spiritual support in palliative care. Research emphasizes that addressing emotional and spiritual needs is crucial for improving patients' overall well-being and QoL. DT’s focus on life review and legacy creation aligns with the broader literature highlighting the value of integrating emotional and spiritual support into palliative care to address patients' complex needs [25].

Impact on Specific Psychological Symptoms

DT is designed to address various psychological symptoms and improve emotional well-being in palliative care patients. The impact of DT on specific psychological symptoms, such as anxiety, depression, and existential distress, reveals both its strengths and limitations. Evaluating these effects is essential for understanding the full scope of DT's therapeutic potential and identifying areas for improvement.

Effects on Anxiety

The impact of DT on anxiety has been inconsistent across studies. For example, Weru et al. [12] found only a trend toward improvement in anxiety, which did not reach statistical significance. This finding suggests that while DT may have some effect on anxiety, it might not be strong enough to produce significant or consistent results in all patients. Conversely, Juliao et al. [13] reported significant reductions in anxiety, indicating that DT can be effective in alleviating this symptom for some individuals. Individual patient characteristics, such as baseline anxiety levels and coping strategies, may influence the effectiveness of DT in reducing anxiety [19].

Effects on Depression

DT has shown more consistent positive effects on depression, though results vary. Juliao et al. [13] observed significant reductions in depression among participants, supporting the view that DT can be an effective intervention for improving mood and emotional state. Similarly, Chochinov et al. [14] reported significant improvements in patients’ sense of dignity and overall well-being, which may contribute to alleviating depressive symptoms. However, Seiler et al. [7] found that DT, when combined with other interventions, did not lead to significant changes in depression, highlighting that the effectiveness of DT on depression may be influenced by factors such as the presence of concurrent therapies and the specific characteristics of the patient population [1].

Effects on Existential Distress

DT’s impact on existential distress has been mixed. Iani et al. [10] reported improvements in meaning and peace but found no significant differences in existential distress or coping ability. This suggests that while DT may help patients find meaning and peace in their experiences, it might not uniformly address existential distress. Karimi et al. [11] also highlighted significant improvements in hope and QoL but did not specifically address existential distress, indicating that DT may have varying effects on different aspects of existential concerns. The variation in outcomes related to existential distress emphasizes the complexity of addressing deep-seated existential issues through DT and suggests that additional support or complementary interventions might be needed [26,27].

Implications and recommendations

Implications

The findings from this systematic review on DT for palliative care patients and their family caregivers reveal several critical implications for both clinical practice and research. The variability in DT’s impact on QoL, psychological well-being, and specific symptoms such as depression and anxiety highlight the need for a nuanced understanding of its effectiveness. While DT shows promise in improving certain aspects of patients’ experiences, its benefits are not universally consistent across all domains or individuals. This suggests that while DT can be a valuable component of palliative care, it should not be viewed as a one-size-fits-all solution. The observed variability highlights the importance of acknowledging that DT may not uniformly address all facets of patients' psychological needs and should be considered as part of a broader, individualized care strategy. Further, the mixed results concerning DT’s effectiveness in managing psychological symptoms, particularly anxiety and depression, highlight the importance of integrating DT with other therapeutic modalities. Combining DT with other evidence-based interventions may enhance overall outcomes and address the diverse needs of patients more comprehensively. For instance, integrating DT with cognitive-behavioral therapy or pharmacological treatments could potentially offer a more robust approach to managing complex psychological symptoms and improving overall patient well-being. Moreover, the variability in DT’s effectiveness emphasizes the need for personalized treatment approaches. Tailoring DT interventions to individual patient needs, including their specific psychological and emotional states, can improve the likelihood of achieving meaningful outcomes. This personalized approach ensures that DT is adapted to address the unique concerns and conditions of each patient, thereby maximizing its relevance and impact. Assessing individual baseline psychological states and incorporating patient preferences into the therapeutic process can lead to more effective and targeted interventions. Additionally, the influence of timing on DT’s effectiveness, as highlighted by studies such as Jenewein et al. [8], suggests that the timing of the intervention plays a crucial role in its impact. Implementing DT at optimal times, based on the patient’s condition and needs, may enhance its effectiveness, and improve overall outcomes. Timing considerations include assessing the stage of illness and the patient’s readiness to engage in DT, ensuring that the therapy is introduced when it can provide the most significant benefits. Lastly, the mixed results and variability in study outcomes point to several research gaps. Further studies are needed to explore the mechanisms through which DT impacts psychological well-being and to identify which patient characteristics are associated with the most significant benefits. This research should also examine how DT can be effectively integrated with other therapeutic approaches to maximize its impact. Additionally, exploring long-term effects and potential benefits for both patients and their families will provide a more comprehensive understanding of DT’s role in palliative care.

Recommendations

Based on the findings of this systematic review, the following recommendations are proposed to enhance the application of DT in palliative care and guide future research efforts. These recommendations address the variability in DT’s effectiveness, suggest ways to optimize the therapy, and highlight areas for further investigation. They aim to improve the impact of DT on patient and caregiver outcomes and ensure that future research continues to refine and advance the use of DT in palliative care settings.

Tailor DT to Individual Needs: Given the observed variability in the effectiveness of DT across different studies, it is essential to individualize DT interventions. This involves a thorough assessment of each patient’s baseline psychological and emotional state to tailor the therapy specifically to their concerns. A multidisciplinary care team, including psychologists, social workers, and palliative care specialists, can collaboratively develop comprehensive care plans that integrate DT with other interventions to address the broad spectrum of patient needs and implement these care plans, ensuring that all dimensions of patient care are addressed. By personalizing DT, healthcare providers can enhance its relevance and impact, ensuring that it addresses the individual needs of patients more effectively.

Integrate DT With Other Therapeutic Modalities: The mixed results regarding DT’s impact on psychological symptoms, such as anxiety and depression, suggest that DT may achieve better outcomes when combined with other therapeutic approaches. Integrating DT with established interventions like cognitive-behavioral therapy or pharmacological treatments can offer a more comprehensive strategy for managing complex psychological issues. This combination could address a wider range of psychological needs and potentially enhance overall patient outcomes.

Optimize Timing and Context for DT: The effectiveness of DT is significantly influenced by its timing and context. It is crucial to administer DT at the most opportune moments, considering factors such as the patient's stage of illness and their readiness to engage in the therapy. By strategically timing the intervention, healthcare providers can maximize its benefits and improve its overall effectiveness. Assessing the patient’s condition and readiness will help ensure that DT is introduced when it can provide the most meaningful support.

Enhance Training and Standardization: To improve the consistency and quality of DT, it is important to enhance the training for healthcare providers administering the therapy. Standardizing the DT protocol and training procedures can help ensure that the therapy is delivered effectively and uniformly across different settings. This can reduce variability in outcomes and improve the overall efficacy of DT.

Focus on Long-Term Effects and Family Caregivers: Future research should investigate the long-term effects of DT on both patients and their family caregivers. Understanding how DT influences long-term well-being and family dynamics can provide valuable insights into its sustained benefits and identify areas for improvement. Additionally, exploring the impact of DT on family caregivers can help understand its broader effects on the caregiving experience.

Address Research Gaps and Variability: To better understand the variability in DT’s effectiveness, future studies should focus on elucidating the mechanisms through which DT impacts psychological well-being. Research should aim to identify patient characteristics that predict significant benefits from DT and explore how DT can be integrated with other therapeutic approaches. Investigating the therapy’s effectiveness in diverse palliative care settings will further clarify its role and impact.

Promote Patient and Caregiver Involvement: Involving patients and caregivers in the DT process can enhance its relevance and effectiveness. By encouraging active participation from patients and caregivers in shaping the therapy according to their preferences and needs, interventions can become more meaningful and impactful. This collaborative approach can also improve satisfaction with the therapy and ensure it meets the specific needs of those receiving it.

Strengths and Limitations

The systematic review presents several strengths that enhance its contribution to understanding DT in palliative care. Firstly, the review benefits from a comprehensive inclusion of studies spanning diverse geographical locations. This wide coverage provides a broad perspective on DT's effectiveness across various cultural and healthcare settings. Additionally, the review's detailed assessment of the risk of bias, as outlined in Table 1, highlights that most studies demonstrate low risk in key domains such as random sequence generation and selective reporting, which strengthens the reliability of the review's conclusions. Furthermore, the review incorporates a diverse range of outcome measures, including QoL, hope, anxiety, depression, and family cohesion, allowing for a thorough evaluation of DT's multifaceted impact on patients and caregivers. The inclusion of recent studies, such as those by Seiler et al. [7] and Jenewein et al. [8], ensures that the review reflects current practices and advancements in palliative care.

However, there are notable limitations in the review. Variability in the methodological quality of the included studies is a significant concern. Several studies exhibit high risk in domains such as blinding and allocation concealment, which can affect the robustness of the review's findings. This variability suggests that the conclusions should be interpreted with caution. Inconsistent reporting and measurement of outcomes across studies further complicate the analysis. For example, while some studies report significant improvements in psychological distress (e.g., Juliao et al. [13]), others show no substantial changes (e.g., Seiler et al. [7]). This inconsistency challenges the ability to draw definitive conclusions about DT's overall effectiveness. Additionally, the review includes studies with relatively small sample sizes and short follow-up periods, such as Iani et al. [10], which limits the generalizability and long-term assessment of DT's effects. The presence of high risk of bias in certain studies such as Iani et al. [10] and Karimi et al. [11], further stresses the need for caution in interpreting the findings. Lastly, the diverse outcome measures used across studies make direct comparison and aggregation of results challenging. Different tools may measure similar constructs in varying ways, affecting the comparability of results. Addressing these limitations in future research could enhance the reliability and applicability of findings related to DT in palliative care.

## Conclusions

DT offers significant benefits in improving certain aspects of quality of life and psychological well-being for palliative care patients. However, its effectiveness varies across different domains and individuals. To maximize the therapeutic benefits of DT, it is essential to integrate it with other interventions, adapt it to individual patient needs, and consider the timing of its application. Further research is crucial to refine and enhance the implementation of DT in palliative care, ensuring that it meets the diverse needs of patients and their families effectively. The variability in DT’s effectiveness emphasizes the need for an adaptive approach to its application, highlighting the importance of individualized care and continued exploration in the field of palliative care.
